# Intestinal Perforation by Tænia Serrata

**Published:** 1897-12

**Authors:** Harri H. Dell

**Affiliations:** Sullivan, Ill.


### INTESTINAL PERFORATION BY TÆNIA SERRATA.

By Harri H. Dell, D.V.S.,
SULLIVAN, ILL.

*Subject:* Gordon setter bitch. *History:* About six weeks previously she had given birth to five puppies. While engaged in her maternal duties, about noon on April 19th, she suddenly evinced a desire to leave the room. Her mute request being granted, she remained out of doors for probably half an hour, but, on being readmitted, appeared to be in great distress, vomiting freely. The family, all cynophilists, in that they lavished their affections upon their canine friends, at once came to the conclusion that some envious neighbor had poisoned the animal, and hastily sent for me.

On arrival, found the animal suffering greatly, temperature 102° F., hurried respiration, pain and tenderness over the abdominal region, percussion of the latter giving a dull sound. Diagnosis, peritonitis with effusion. Prognosis unfavorable.

Ordered hot applications to abdominal region and left sedatives to be administered at short intervals, which, while affording some relief, could not stay the inevitable progress of the pathological conditions, death occurring the following day at noon.

I was eager for, yet somewhat fearful of, an autopsy; for, to my mind, while there was undoubtedly a subacute or chronic peritonitis, the fact that the animal had not shown any unusual symptoms until the day preceding death seemed not to warrant the diagnosis.

The autopsy was held three hours after death, revealing the following conditions: On opening the abdominal cavity it was found to contain three pints of a thick, reddish sero-fibrinous fluid, floating in which were numerous segments, varying in length, of some variety of tænia. There was a diffuse peritonitis involving the parietal and visceral layers. The intestinal folds contracting the stomach were united by adhesions of organized exudate. The gastric omentum was much thickened as a result of the inflammatory processes, showing many areas of necrosis; attached firmly to the more healthy portions were the cephalic ends of seven tænia. Siphoning off the fluid, a close search for perforation of the intestine was rewarded by finding three in number about four centimetres in diameter, the edges roughened and brown in color. The stomach contained a small quantity of partly-digested milk, the mucosa pale and evidencing no inflammatory changes. The duodenum and jejunem contained semi-digested food, the mucous coat of the ileum showing circumscribed areas of inflammation, adhering to which were the cephalic ends of seven tænia. The perforations were through the walls of this portion of the tract. The kidneys were slightly congested, the bladder containing a small quantity of urine. Examination of the thoracic organs revealed nothing worthy of note.

Pathological diagnosis: Perforating wound of the intestine, diffuse sero-fibrous peritonitis, due to the presence of tænia serrata, possibly complicated by the entrance into the peritoneal cavity of septic material from the intestine, traumatic cellular proliferation of the mesentery and gastric omentum, sero-fibrinous exudate and adhesions of the intestinal folds to the stomach; death resulting from septic infection and cardiac failure.

The intestinal walls were not thinner than normal; if anything, they were slightly thicker, and those portions surrounding the perforations exhibited no evidence of ulceration past or present, forcing the conclusion upon the writer that the migration of the tænia was quite voluntary on their part.

Among the peculiar features of the case is the fact that the owners had noticed no symptoms that indicated the extensive pathological alterations which were taking place, for undoubtedly the migration had been effected a considerable time before medical assistance was sought.

Intestinal perforation by tape-worms must indeed be rare, for in several hundred autopsies on dogs (in few of which tænia of some form were absent), this is the first case to come under my notice. Menin, however, records a case in the horse, with the formation of diverticula and cysts. In the *Revue Veterinaire*, 1888, M. Cadeac records the case of a terrier exhibiting symptoms simulating those of rabies. On being destroyed, a perforation of the duodenum was found, engaged in which was a tænia (species not given), another one being free in the peritoneal cavity. This is the only other case in the dog I have been able to discover in the literature at my disposal; but through the courtesy of Dr. Cecil French, who has very kindly searched the libraries of the Army Medical Museum and the Bureau of Animal Industry, I am enabled to add still another case. In the *Receuil Veterinaire*, 1888, Lahogue reports a case exhibiting rabiform symptoms, the autopsy revealing three perforations due to tænia serrata, but recording no other lesions. It will thus be seen that both these Continental cases differed in the symptoms from the case under discussion, the duties of the maternal condition in the latter not being sufficient to account for the insidious nature of the pathological changes.

